# Characteristics and outcomes of pediatric oncology patients at risk for guardians declining transfusion of blood components

**DOI:** 10.1002/cnr2.1665

**Published:** 2022-07-06

**Authors:** Jason Stevenson, Nicholas P. DeGroote, Frank Keller, Katharine E. Brock, D. John Bergsagel, Tamara P. Miller, Patricia Cornwell, Ross Fasano, Satheesh Chonat, Sharon M. Castellino

**Affiliations:** ^1^ Department of Pediatrics, Division of Graduate Medical Education Emory University Atlanta Georgia USA; ^2^ Aflac Cancer and Blood Disorders Center, Children's Healthcare of Atlanta Atlanta Georgia USA; ^3^ Department of Pediatrics, Division of Pediatric Hematology/Oncology Emory University Atlanta Georgia USA; ^4^ Department of Pediatrics, Division of Pediatric Palliative Care Emory University Atlanta Georgia USA

**Keywords:** Jehovah's witness, pediatric oncology, transfusion refusal

## Abstract

**Background:**

Transfusion of blood products is a necessary part of successful delivery of myelosuppressive regimens in pediatric cancer. There is a paucity of literature characterizing outcomes or management of pediatric patients with cancer when transfusion is declined.

**Aims:**

The objective of this paper is to describe the clinical characteristics, care, and outcomes of patients with cancer at risk for declining transfusion.

**Methods and Results:**

A retrospective cohort of patients aged 0–21 years with cancer managed at Children's Healthcare of Atlanta between 2006 and 2020 and with ICD‐9 codes indicating risk of “transfusion refusal” or Jehovah's witness (JW) religion was identified. Demographics, disease, and management were abstracted. Descriptive statistics were performed to examine associations with transfusion receipt. Among 35 eligible patients identified as at risk for declining transfusion, 89% had primary guardians who identified as JW, and 45.7% identified as Black, non‐Hispanic. Only 40% of guardians actively declined transfusion. Transfusion recipients had significantly lower hemoglobin (g/dl) and platelet counts (1000/μl) at initial presentation (9.6 vs. 11.9, *p* < .002 and 116.0 vs. 406.5, *p* = .001, respectively) and at nadir (5.9 vs. 8.7, *p* < .001 and ≤ 10 vs. 154, *p* < .001, respectively) than non‐recipients. Legal intervention was required in 36.4% of those who ultimately received a transfusion.

**Conclusion:**

Among pediatric cancer patients whose medical record initially indicated a preference for no transfusion, 60% of guardians accepted blood products when prescribed for oncology care. Guidelines for systematic management and transfusion sparing approaches are needed to honor guardian's preferences when possible yet while maintaining equitable cancer outcomes in this population.

## 1 INTRODUCTION

Disease outcomes in pediatric patients with cancer have improved significantly in part due to increasingly myelosuppressive chemotherapy regimens.^1^, ^2^ Transfusion of blood products is a key element of supportive care that facilitates successful delivery of myelosuppressive regimens. In adult patients who decline transfusion, providers must uphold the principle of respect for autonomy.^3^ When medical providers deem that transfusions are necessary and when non‐emergent medical needs allow planning, blood sparing approaches such as acute hypervolemic hemodilution, cell saver techniques, and erythropoiesis stimulation in advance of procedures can be used in lieu of allogenic blood product transfusion.^3^ However, when guardians of pediatric cancer patients refuse or “decline” transfusion, the medical provider is faced with unique medical, legal, and ethical challenges. Current literature regarding declining transfusion in pediatric oncology patients is limited to small case series and lacks specificity about clinical characteristics, management, and outcomes.^3^, ^4^, ^5^, ^6^, ^7^

Pediatric patients under 18 years of age, or older patients under a guardian's care for medical decisions, lack the ability to legally provide consent for cancer treatment or associated supportive care. Based on legal precedent set by multiple court cases in the United States, the government retains the power to intercede and act in the best interest of the child.^8^, ^9^ This doctrine, known as *parens patriae*, is applied in situations in which treatment is deemed reasonable and necessary.^8^ Court cases specific to the issue of declining blood transfusion by a parent, such as the 1991 case of Elisha McCauley, have ruled that despite the parents' right to religious freedom, the best interest of the child outweighs the parents' objections when a child's life is at stake.^9^ While legal intervention is possible in most cases, the process risks stressing the therapeutic relationship between the medical team, the patient, and the patient's caregivers and may result in mistrust of both the provider and medical system. This added tension may further expose implicit and explicit biases in decision making by the medical team.^10^ More importantly, pursuing legal intervention can delay timely delivery of critical cancer treatment. As pediatric cancer treatment and care can span several years, maintaining a therapeutic alliance is crucial to successful delivery of therapy that gives all children the best chance at positive and equitable outcomes.^4^, ^5^, ^11^

Review papers on declining transfusion are limited by the lack of information on children or adolescents with cancer.^3^, ^6^, ^7^ No national or society guidelines exist for cases when guardians decline transfusion, and there is a paucity of literature characterizing management and outcomes of pediatric patients with cancer in this situation. The aim of this study is to describe the clinical characteristics, supportive care, and outcomes of children, adolescents, and young adults at risk for declining transfusion during cancer therapy.

## 2 METHODS

A retrospective chart review was conducted at the Aflac Cancer and Blood Disorders Center at Children's Healthcare of Atlanta (CHOA). All children aged 0–21 years who were treated at CHOA for de novo or relapsed cancer between January 1, 2006 and January 1, 2020 with the electronic medical record indicated a risk for declining transfusion were eligible for inclusion. Patients who initiated treatment at a different institution or who lacked detailed health records were excluded from the study. The study was reviewed and approved by the CHOA Institutional Review Board.

Patients with an oncologic diagnosis in the institutional cancer registry at risk for declining transfusion during the study time period were identified through three methods: (1) ICD‐9 and ICD‐10 codes from the electronic medical record (EMR) indicating potential risk factors for declining transfusion or documented occurrences of an event of declining transfusion; (2) patient lists maintained by CHOA providers, cases managers, social workers, and disease‐based teams for clinical purposes; and (3) EMR review search terms including “transfusion refusal” or “Jehovah's witnesses” (JW). The EMR was queried for the entirety of patients listed in the CHOA cancer registry.

The following ICD‐9 codes were included in review: V62.6 (Refusal of treatment for reasons of religion or conscience) and V62.89 (Other psychological or physical stress, not elsewhere classified [Synonym: Religious or spiritual problem]). In addition, the ICD‐10 codes included: Z53.1 (Procedure and treatment not carried out because of patient's decision for reasons of belief and group pressure [Synonym: Patient declines blood products for reasons of religion or conscience or Transfusion of blood product refused for religious reason]) and Z78.9 (Other specified health status [Synonym: Patient is Jehovah's Witness]).

Study variables were manually abstracted from the EMR and included demographics, disease factors, hematologic laboratory values, supportive care medications, disease management, and the type and date of transfusion product delivered. Clinical characteristics included cancer type (hematologic malignancy, solid tumor, or central nervous system [CNS] tumor), serum hemoglobin (Hgb) (g/dl) at presentation, platelet count (1000/μl) at presentation, and Hgb or platelet count nadir throughout the course of therapy. Outcomes and interventions captured included vital status (alive/deceased) at date of abstraction (December 2020), enrollment in a therapeutic clinical trial, admission to the intensive care unit (ICU), active declining of blood products, receipt of erythropoietin (EPO), need for legal support or intervention, and receipt of an ethics consult. EMR notes were reviewed for text suggesting cancer therapy modifications associated with guardian's preference to decline transfusion. The main outcome of interest was receipt and date of a blood product including packed red blood cells (PRBC), platelets, fresh frozen plasma (FFP), cryoprecipitate, or intravenous immunoglobulin (IVIG).

Descriptive statistics were conducted on all study variables including frequencies and percentages for categorical variables and medians and interquartile ranges (IQR) for non‐parametric numeric variables. Independent two‐sample *t* tests and Chi‐square/Fisher's exact tests were used to assess differences in demographics, clinical factors, and other outcomes by receipt of blood products. *p*‐values were two‐sided and considered statistically significant if *p* < .05. SAS Enterprise Guide 7.1 (Cary, NC) was used for all analyses.

## 3 RESULTS

**FIGURE 1:**
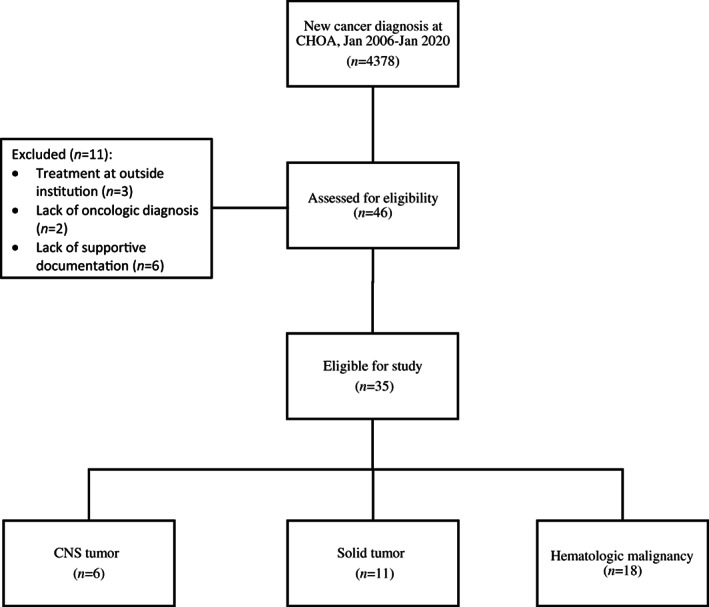
Consort diagram of patient eligibility

Forty‐six patients with a new cancer diagnosis were initially identified as potentially eligible among 4378 patients with a new cancer diagnosis at the institution during the study period. Eleven patients were excluded due to receipt of treatment outside institution, lack of oncologic diagnoses, and lack of adequate EMR documentation in provider notes about decline of blood transfusion or risks of decline of blood transfusion. In total, 35 patients met inclusion criteria and were subsequently included in the study cohort (Figure 1). Overall, 63% (22/35) of patients were male, 46% (16/35) identified as Black, non‐Hispanic, and 25% (9/35) were Hispanic/Latino. The median age of the child at diagnosis was 8 years (IQR: 4, 20 years). Most (31/35, 89%) primary guardians identified themselves as JW. Diagnoses included hematologic malignancies (51%, 18/35), solid tumors (31%, 11/35), or CNS tumors (17%, 6/35) (Table 1).

**TABLE 1:** Characteristics of pediatric oncology patients at risk for declining blood transfusion (*N* = 35)

	Overall (*N* = 35)	
Age at diagnosis, years (median/IQR)	8.0	(4, 20)
	*N*	%
**Sex**		
Male	22	62.9
**Race/ethnicity**		
White, non‐Hispanic	7	20.0
Black, non‐Hispanic	16	45.7
Other/not specified, non‐Hispanic	3	8.6
Hispanic/Latino	9	25.7
**Insurance**		
Private	9	25.7
Medicaid	18	51.4
Other/not specified	8	22.9
**Religion**		
Jehovah's witnesses	31	88.6
Other Christian denomination	4	11.4
**Diagnosis**		
Hematologic malignancy	18^a^	51.4
Solid tumor	11	31.4
CNS tumor	6	17.1

Abbreviations: CNS, central nervous system; IQR, interquartile range.

^a^ One patient initially diagnosed with osteosarcoma later developed secondary leukemia.

Forty percent (14/35) of patient's guardians actively declined a blood product transfusion. Two patients declined transfusion and did not receive a blood product transfusion. No patients over the age of 18 (*n* = 2) declined blood product transfusion. Overall, 22/35 (62.8%) patients required and received at least one blood product (Table 2). Among the patients who received a transfusion, 18% (4/22) of guardians initially declined to consent for blood transfusion, but subsequently allowed transfusion. Nine patients who received a transfusion did not actively decline transfusions and did not require legal intervention. Legal intervention was required for transfusion in 36.4% (8/22) who received transfusion, and an ethics consult was sought and completed for one patient. Among patients who required legal intervention, all received a blood transfusion.

**TABLE 2:** Demographics among pediatric oncology patients at risk for declining transfusion stratified by receipt of transfusions (*n* = 35)

	Received blood products (*N* = 22)		No blood products (*N* = 13)		*p*‐value
	*N*		*N*		
**Age at diagnosis, years (median/IQR)**	4	(2, 12)	16	(8, 16)	.02
		**%**		**%**	
**Sex**					
Male	16	72.7	6	42.9	.17
**Race/ethnicity**					
White, non‐Hispanic	3	13.6	4	42.9	.77
Black, non‐Hispanic	11	50.0	6	28.6	
Other/not specified, non‐Hispanic	2	9.1	1	28.6	
Hispanic/Latino	6	27.3	3		
**Insurance**					
Private	5	22.7	4	30.8	.48
Medicaid	13	59.1	5	38.5	
Other/not specified	4	18.2	4	30.8	
**Religion**					
Jehovah's witnesses	18	81.8	13	100.0	.27
Other Christian denomination	4	19.1	0	0.0	
**Legal**					
Legal intervention	8	36.4	0	0.0	.02

Abbreviation: IQR, interquartile range.

**FIGURE 2:**
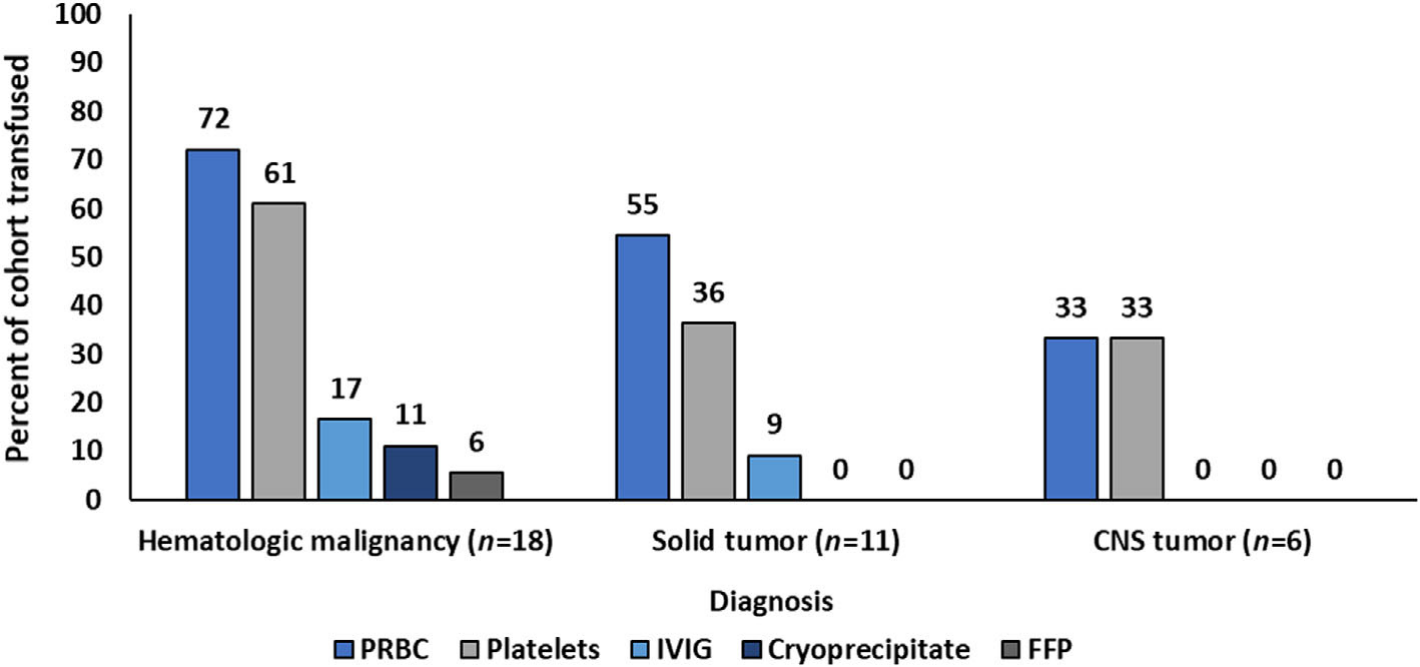
Blood products administered by cancer diagnosis among pediatric patients at risk for declining transfusion (*n* = 35)

Younger patients were significantly more likely to receive blood products (*p* = .02) (Table 2). Patients received the following products: PRBC (21/22, 95.5%), platelets (17/22, 77.3%), IVIG (5/22, 22.7%), cryoprecipitate (2/22, 9.1%), and FFP (1/22, 4.6%) (Table 3). Cryoprecipitate and FFP were given in the setting of diffuse intravascular coagulation. Among transfusion‐recipients, 59.1% (13/22) had a hematologic malignancy. Transfusion recipients had significantly lower Hgb (g/dl) and platelet (1000/μl) counts at initial presentation (Hgb 9.6 vs. 11.9, *p* = .002 and platelets 116.0 vs. 406.5, *p* = .001) and at nadir (Hgb 5.9 vs. 8.7, *p* < .001 and platelets ≤10 vs. 154, *p* < .001) than non‐recipients (Table 3). Among the 31 patients who received chemotherapy, 9.7% (3/31) had treatment modifications noted in the consent conference documentation to reduce the risk of need for transfusion. Adjunct supportive care included erythropoietin with a starting daily dose of 150 units/kg in 10 patients (5 of whom never received a transfusion) and ferrous sulfate at 4–6 mg/kg/day in 7 patients (3 of whom never received a transfusion). Of the 10 patients that received erythropoietin, 6 also received iron with only 1 patient receiving isolated iron therapy. None of the 10 patients that received erythropoietin had thromboembolic or cardiovascular events. Anti‐fibrinolytic management included aminocaproic acid in five patients; no patients received tranexamic acid. There were no significant differences in ICU admissions (40%, 14/35) or enrollment on a therapeutic clinical trial (37.1%, 13/35) by receipt of blood products. Overall, 62.8% of patients received blood products (22/35). More patients with a hematologic malignancy received at least one transfusion (13/18, 72.2%) compared with solid tumor (6/11, 54.6%) and CNS tumor (3/6, 50.0%) patients, though this difference was not statistically significant (*p* = .46). The most common blood products by hematologic malignancy, solid tumor, and CNS tumor were PRBCs (72%, 55%, and 33%, respectively), platelets (61%, 36%, and 33%, respectively), and IVIG (17%, 9%, and 0%, respectively) (Figure 2). Eight patients are deceased; the median time from diagnosis to death was 333.5 days (range 61–1281). Of the eight deceased patients, seven received a blood transfusion. Deaths were not directly attributed to severe anemia or thrombocytopenia.

**TABLE 3:** Clinical outcomes among pediatric oncology patients at risk for declining transfusion stratified by receipt of transfusions (*n* = 35)

	Received blood products (*N* = 22)		No blood products (*N* = 13)		*p‐*value
**Diagnosis**	***N***	**%**	***N***	**%**	
Hematologic malignancy (*n* = 18)	13	59.1	5	38.5	.46
Solid tumor (*n* = 11)	6	27.3	5	38.4	
CNS tumor (*n* = 6)	3	13.6	3	23.1	
**Hemoglobin (g/dl)**	N	(median, IQR)	N	(median, IQR)	
Presentation	9.6	(7.3, 11.2)	11.9	(11.1, 14.3)	.002
Nadir	5.9	(5.3, 6.4)	8.7	(8.4, 9.9)	<.001
Prior to first transfusion	6.7	(6.2, 7.4)			
**Platelet count (1000/μl)**	*N*	(median, IQR)	*N*	(median, IQR)	
Presentation	116.0	(45.0, 322.0)	406.5	(309.0, 528.0)	.001
Nadir	≤10^a^	(≤10.0, 16.0)	154	(88.5, 178.0)	<.001
Prior to first transfusion	14^a^	(≤10.0, 24.0)			
**Supportive therapy**					
EPO	5	22.7	5	37.1	.44
Aminocaproic acid	5	22.7	0	0.0	.13
Ferrous sulfate	4	18.2	3	23.1	1
Iron sucrose	2	9.1	0	0.0	.51
Vitamin B12	0	0.0	1	7.7	.37
**Number of administrations by blood product**					
PRBC	21	95.5			
Administrations (median, IQR)	5.0	2, 7			
Platelets	17	77.3			
Administrations (median, IQR)	6.0	4, 9			
IVIG	5	22.7			
Administrations (median, IQR)	2.0	1, 6			
FFP	1	4.6			
Administrations (median, IQR)	7.0	7, 7			
Cryoprecipitate	2	9.1			
Administrations (median, IQR)	2.0	1, 3			

*Note*: Three patients excluded: one received blood products 271 days prior to hematologic malignancy diagnosis due to idiopathic thrombocytopenia purpura, two received blood products within 1 week prior to oncologic diagnosis.

Abbreviations: CNS, central nervous system; EPO, erythropoietin; FFP, fresh frozen plasma; IQR, interquartile range; IVIG, intravenous immunoglobulin; PRBC, packed red blood cells.

^a^ Platelet count <10 in the EMR were recorded as 10.

## 4 DISCUSSION

Declining transfusion of blood products in the setting of critical anemia or thrombocytopenia limits therapy delivery and risks significant morbidity and mortality in children with cancer.^12^ We present the largest series to date of children and adolescents with cancer at risk for declining transfusion. Overall, 1% of patients cared for within a single pediatric cancer center were identified as being at risk for declining transfusion. However, we observed that active guardian refusal of transfusion occurred in only 40% (14/35) of families and largely impacted children with hematologic malignancies. While our methodologic approach broadly searched for ICD‐9/10 codes indicating transfusion decline not specific to religion, cases of declining transfusion were largely associated with parental identification as JW faith.

In pediatric oncology, parental decline of transfusion is most commonly seen in children of JW parents. JW number over 300 000 within the United States and 8.6 million worldwide.^13^, ^14^ In the JW faith, interpretation of biblical verse prohibits autologous or allogenic transfusions but leaves the decision regarding use of cryoprecipitate and immunoglobulins to the individual, as they are considered minor blood fractions.^2^, ^15^, ^16^, ^17^, ^18^ Our data in children is consistent with prior literature in adult patients indicating that under anonymity, 10%–12% of people identifying as JW are willing to accept blood products for themselves.^7^ In children, the requirements for parent/legal guardian consent and many hospital system's requirement for written consent at each transfusion encounter could contribute to parental distress in cases where there is a faith‐based tenet to not receive blood products. Medically, ethically, and legally, the urgent nature of a cancer diagnosis and specifically of a cancer diagnosis in a child who could benefit from timely and efficacious treatment makes this situation unique from cases afforded planning time before anesthesia or elective surgery.^7^ Beyond the risk of morbidity and mortality, declining of transfusions in pediatric oncology may delay life‐saving treatment, threaten parental trust of the medical system, uncover provider biases in care, and disrupt the patient‐family‐provider relationship.^12^ Notably, in our series a large portion of patients (71%) identified as people of color. Recent literature has called for consideration of oncologic care in patients of minority race and ethnicity through the lens of the principles of beneficence, nonmaleficence, and justice, and asks providers to consider implicit biases in decision making.^19^, ^20^ Patients who are at risk of declining transfusion represent an under‐represented population for whom providers need to weigh these ethical principles toward the goal of equity in care and outcomes for the child and the family.

Many institutions lack systematic guidelines for addressing these time sensitive situations. While our children's hospital does not have a formal policy or guidelines for the management of blood product administration to patients declining transfusions, we follow a pragmatic approach on a case‐by‐case basis. Discussions involve the patient, family, social worker, JW liaison, and hospital legal team liaison. Depending on the level of anemia, cardiorespiratory status, and clinical urgency, the family is notified that a court order may be sought to provide immediate life‐saving blood products. While medical providers might view these differences as challenging the standard decision model, a clear understanding of parent wishes and which blood products are not acceptable, as well as inclusive discussions involving JW parents and members of the JW community (i.e., JW Elders), could increase effective communication and understanding. In the future, the availability of consensus guidelines could mitigate the risk of bias on clinical care.

Only 37% of patients at risk for declining transfusion were enrolled on a clinical trial, with the small sample size making it difficult to know whether trial eligibility or availability was an issue, or whether implicit bias by the provider was involved, given that supportive care with transfusion is standard of care for therapy intensive chemotherapy trials.^21^

While providers consider transfusion a standard medical procedure, blood transfusions were viewed as harmful by up to 10.9% of patients and 14.6% of caregivers.^22^ Concerns included the risk of adverse reactions and infection.^22^ Because caregivers may consider receipt of donor blood an invasive medical intervention, thoughtful care communication should focus on mutual goals for the child while addressing parental worries and preserving the therapeutic relationship.^23^

Despite our broad inclusion criteria, no other cultural or religious identification other than that of JW faith was noted among our cohort. Our results indicate that the majority of guardians ultimately accepted blood product transfusions when needed for the child's care and legal intervention was only required in 8 patients (23%) overall. Of the 8 patients in which legal intervention was required, 5/8 (62.5%) had a diagnosis of a hematologic malignancy. Therefore, providers should not assume that all JW patients will decline transfusions, and empathic listening and engaged conversation will be crucial in each case.

The lack of uniform guidance may lead to non‐standard cancer treatments, such as the use of alternative, less myelosuppressive chemotherapeutic agents or dose reductions; these modifications risk disparate outcomes. Adjustments to chemotherapy regimens and adjunctive therapies, such as erythropoiesis stimulating agents, ferrous sulfate, folic acid, and antifibrinolytics may attenuate the need for transfusion. However, these approaches take time and in many clinical circumstances in pediatric oncology, timely diagnostic procedures and treatment is paramount. In addition, there are limited studies to determine the effect transfusion sparing adjunctive therapies may have on outcomes, including disease free survival.^24^, ^25^, ^26^, ^27^, ^28^, ^29^

The routine use of erythropoietin stimulating agents in cancer is controversial, with limited data in children with cancer.^28^ The association of these agents with thromboembolic and cardiovascular events, increased tumor growth, and increased mortality, lead to an FDA black box warning.^27^ Prior case series have indicated that the use of erythropoietin and iron as supplemental therapy during pediatric cancer treatment is safe and may increase baseline hemoglobin and reduce the need for transfusions.^5^, ^24^, ^25^, ^26^, ^27^ Neither erythropoietin nor iron were systematically used in our cohort with only 28.6% of patients receiving erythropoietin therapy and 20% receiving iron therapy. Contrary to the findings by Bohlius et al., there were no noted thromboembolic or cardiovascular events noted with the use of erythropoietin in our cohort.^27^

In evaluating feasibility of delivering intensive cancer therapy Tenenbaum et al. evaluated the use of supportive measures including iron, erythropoietin, IL‐11, G‐CSF, and stem cell rescue in 14 pediatric oncology patients of JW faith.^5^ The transfusion thresholds on this study included clinically significant anemia or Hgb less than 4 g/dl or platelet counts less than 10 000/μl. Patients tolerated lower hemoglobin and platelet counts without significant issues, leading to the conclusion that JW's can be treated with the same intensive protocols.^5^ In our study, only 3/31 (9.7%) patients who received chemotherapy had their treatment regimens altered upfront with substitution of protocol chemotherapeutic agents to less myelosuppressive agents.

Antifibrinolytics, specifically tranexamic acid and aminocaproic acid, have been used as blood sparing strategies in patients who decline transfusions. Clinical trials in adults have shown that tranexamic acid lessens blood loss without an increased risk of thrombosis.^12^ While these agents may reduce the need for transfusion prior to surgical interventions, anti‐fibrinolytic management was not widely used in our cohort, with only five patients receiving aminocaproic acid, no patients receiving tranexamic acid, and no patients receiving aminocaproic acid prior to surgery for bleeding prophylaxis.

Despite the promise of blood substitutes, development of a successful agent has been challenging due to poor efficacy and toxicities.^30^ Currently, no hemoglobin‐based oxygen carriers (HBOCs) are FDA approved for human use in the United States.^31^ Hemopure (HBOC‐201) has shown promising results in adult patients with sickle cell disease, but use is currently limited in the US for use in adults through investigational or expanded access (NCT02684474).^31^, ^32^ Of note, our pediatric institution did not use any blood substitution products between January 1, 2006 and January 1, 2020 (*personal communication blood bank, RF*).

Limitations of our study include a small sample size obtained from a single institution retrospective analysis. As the total number of oncology patients that require blood product transfusion is unknown, we cannot compare our outcomes to the overall pediatric oncology population. In the event that blood conserving approaches (minimizing phlebotomy incidence and volumes) were used, this was not assessable by our EMR review. In addition, there is a possibility that eligible patients were not included based on our search algorithm.

Despite these limitations, our study is the first to systematically identify a cohort at risk for refusal of transfusion, and to characterize the full spectrum of management and outcome. This study highlights the need for guidelines with stratification for hematologic malignancies versus solid and CNS tumors, as patients with hematologic malignancies often have more urgent needs for transfusions and initiation of therapy. Hematologic malignancies required more transfusions when compared to solid and CNS tumors likely due to a combination of disease processes (including involvement of the bone marrow) as well as duration and intensity of therapy.

Systematic medical‐legal protocols for pediatric oncology patients who decline, or are at risk for declining, transfusion may help to standardize provider approach with the goal of timely, high quality, and goal‐concordant care. These standardized operating procedures may include quality improvement initiatives such as triggering consults with social work at time of diagnosis, limiting frequency and volume of lab acquisition, and early initiation of adjunctive treatment. System policies to accommodate family distress, such as covering of administered blood products should be consistently offered by all provider teams in the hospital. Partnering with the JW community in the development of these protocols and guidelines is necessary to provide high‐quality care for this patient population. Further studies on children with cancer and blood disorders whose caregivers declined transfusions will aid in determining adverse events and outcomes as well as the establishment of objective protocols and guidelines. Subsequent interventional trials of supplemental therapy and artificial blood products are needed to determine the risks and benefits in this pediatric oncology population.

## AUTHOR CONTRIBUTIONS

All authors had full access to the data in the study and take responsibility for the integrity of the data and the accuracy of the data analysis. *Conceptualization*, J.S., N.P.D, S.C., and S.M.C.; *Investigation*, J.S.; *Data curation*, J.S. and N.P.D.; *Writing‐original draft and revisions*, J.S. and S.M.C.; *Methodology*, N.P.D and S.M.C.; *Formal analysis*, N.P.D and S.M.C.; *Writing‐review and editing*, F.K., K.E.B., D.J.B., T.P.M., P.C., R.F., S.C.; *Resources*, F.K. and S.M.C.; *Supervision*, S.M.C.; *Resources writing*, P.C.

## CONFLICT OF INTEREST

The authors have no conflicts of interest or financial relationships relevant to this article to disclose.

## CODE AVAILABILITY

All software application or custom code support the published claims and comply with field standards. Code available upon reasonable request.

## ETHICS STATEMENT

The Children's Healthcare of Atlanta Institutional Review Board reviewed and approved the study.

## Data Availability

All data and materials support the published claims and comply with field standards. Data available upon reasonable request.
